# A prospective, single-centre, single-arm, open label study of the long term use of a gonadotropin releasing hormone agonist (Triptorelin SR, 11.25 mg) in combination with Tibolone add-back therapy in the management of chronic cyclical pelvic pain

**DOI:** 10.1186/s12958-020-00586-z

**Published:** 2020-04-14

**Authors:** Sallwa M. Alshehre, Sheila Duffy, Georgina Jones, William L. Ledger, Mostafa Metwally

**Affiliations:** 1 Academic Unit of Reproductive and Developmental Medicine, The University of Sheffield, The Jessop Wing, Tree Root Walk, Sheffield, S10 3HY, Sheffield, UK; 2 Department of Obstetrics and Gynaecology, The Jessop Wing, Tree Root Walk, Sheffield, S10 3HY UK; 3 School of Social Sciences, Leeds Beckett University, City Campus, Leeds, LS1 3HE UK; 4 The University of New South Wales, Royal Hospital for Women, Barker Street, Randwick NSW, Sydney, 2031 Australia

**Keywords:** Endometriosis, Chronic cyclic pelvic pain, GnRH, Add back therapy

## Abstract

**Background:**

Chronic cyclic pelvic pain (CCPP) affects women’s quality of life and pituitary downregulation is often used for symptomatic relief. However, prolonged suppression of ovarian function is associated with menopausal side effects and can lead to osteoporosis. Currently, the use of gonadotropin releasing hormone agonists (GnRHa) for treatment of CCPP is usually restricted to 6–9 months, limiting their efficacy. There is limited information regarding safety and efficacy with longer-term use. The aim of this study is to examine the safety and efficacy of long-term (24 months) pituitary down-regulation with the GnRHa (Triptorelin SR) with add-back therapy (ABT) using Tibolone for symptom relief in women with CCPP.

**Methods:**

A single-arm, prospective clinical trial at a Tertiary University Teaching Hospital of 27 patients receiving Triptorelin SR (11.25 mg) and Tibolone (2.5 mg). Outcomes measures were the safety of treatment assessed by clinical examination, haematological markers, liver and renal function tests and bone mineral density (BMD) at 12, 18 and 24 months as well as at 6 months post-treatment. Pain and health-related quality of life (HR-QoL) assessed using the endometriosis health profile (EHP-30) and chronic pain grade (CPG) questionnaires.

**Results:**

There was no evidence for any significant harmful effects on any of the measured haematological, renal or liver function tests. Although results regarding the effect on BMD are not conclusive there is an increased risk of development of osteopaenia after 12 months of treatment. Pain and HRQoL assessments showed significant improvement during medication, but with deterioration after treatment cessation.

**Conclusion:**

Long- term Triptorelin plus Tibolone add-back therapy in women suffering from CCPP does not appear to be associated with significant serious adverse events apart from the possibility of deterioration in the BMD that needs to be monitored. This mode of therapy appears to be effective in pain relief and in improving quality of life over a 24-month period.

**Trial registration:**

Clinical trials database NCT00735852.

## Background

Chronic pelvic pain (CPP) is a common problem that affects many women. Estimates of prevalence vary between 4.0 and 43.4% of women globally [1, 2]. CCPP is considered to be one of the most debilitating conditions affecting women of childbearing age [3] and can have a negative impact on a women’s quality of life and ability to function, causing significant loss of time from work, psychological distress and impaired sexual function [4].

In cases of chronic cyclic pelvic pain (CCPP) the pain is synchronous with the menstrual cycle and is usually related to the cyclic fluctuations in ovarian hormones. Often such pain may be a result of gynaecological conditions such as endometriosis or adenomyosis but in some instances the pain can be non-gynaecological in nature. For instance, CCPP can be the result of neurological or musculoskeletal causes [5–7], gastrointestinal tract conditions such as irritable bowel syndrome [8] or urinary tract conditions such as interstitial cystitis [9]. In many cases, there is no clear diagnosis, presenting a clinical dilemma and limiting the options that can be offered.

In determining the most effective treatment for chronic cyclical pelvic pain and endometriosis, many factors must be considered including the severity of symptoms and effect on quality of life (QoL), the feasibility of surgery and associated surgical risk, the patient’s need for future fertility and tolerance to treatment side effects [10].

Pituitary downregulation with gonadotropin releasing hormone agonists (GnRHa) is often used to treat women with severe CCPP who are not trying to conceive [11, 12], where simple analgesics and progestogen therapy are not effective and where surgical intervention is not feasible, deemed too high risk or not the patient’s preference.

Prolonged administration of GnRHa initially causes pituitary stimulation followed within a few days by pituitary desensitisation due to persistent occupation of GnRH receptors by the long-acting synthetic GnRHa, preventing normal receptor recycling. This results in a rapid decrease in the circulating concentrations of FSH and LH [13] with profound suppression of ovarian steroid secretion within two weeks of starting treatment [14].

A maximum of 6 months of treatment with GnRH analogues such as Triptorelin is currently licensed for ovarian downregulation [15, 16]. This is due to the potential for reduction in bone mineral density (BMD) that follows prolonged periods of hypo-oestrogenisation [13, 17]. The rate of loss of bone density is in the range of 0.5–1.0% per month, which over time increases the risk of osteoporosis [18]. Restriction of use of GnRHa to 6–9 months severely limits clinical efficacy: no sooner has the patient derived maximum benefit from the treatment than she is advised to stop. However, several studies have shown the efficacy of hormone replacement “addback” therapy prescribed in conjunction with GnRHa to reduce the impact of low circulating oestrogens on bone density. The principle of addback therapy is based on the “oestrogen threshold” hypothesis, aiming to increase circulating oestrogen concentrations to a level sufficient to sustain bone integrity and prevent menopausal side effects but not sufficient to stimulate growth of endometrial tissue or cause recurrence of pain [16, 18]. Many patients with severe CCPP respond well to GnRHa but experience rapid symptom recurrence on cessation of treatment, and would benefit from prolongation of therapy [19]. Clinical data regarding the long-term safety and efficacy of GnRHa beyond 6 months is currently lacking.

In this study, we therefore examine the safety and efficacy of prolonged ovarian downregulation for 24 months using the GnRH, Triptorelin in combination with Tibolone addback therapy. Tibolone is a synthetic molecule which combines oestrogenic, progestogenic and weak androgenic actions and is commonly used for addback with GnRHa [20].

We assessed the efficacy of this combination therapy used over a 24-month period, studying the effects on both chronic pelvic pain symptoms and quality of life along with regular assessment of BMD and other markers of clinical safety.

## Methods

The study was a single-centre, single-arm, open-label prospective clinical trial to evaluate the efficacy and safety of extended treatment (24 months) with Triptorelin (Gonapeptyl® SR) 11.25 mg every three months administered in combination with Tibolone (Livial®) tablets 2.5 mg daily for the treatment of women with CCPP. As the study included within-patient comparisons, there was no control arm and all patients received the same active treatment. Thirty-one premenopausal patients aged 20–45 with CCPP who attended the gynaecology clinic at the Jessop Wing, Sheffield Teaching Hospitals, Sheffield, UK, and who satisfied the inclusion/exclusion criteria were recruited for the study from December 2008 with the last participant completing the study in May 2016.

Women were included if they were aged between 18 and 45 years inclusive with a clinical diagnosis of CCPP defined as suffering from pelvic pain, strongly related to the menstrual cycle, severe enough to interfere with daily activity and of at least 6 months duration (with or without endometriosis). Participants must have had investigations for possible endometriosis within three years prior to the screening visit (laparoscopy or ultrasound). All women must have had regular menstrual cycles (between 24 and 42 days) for the 3 months prior to screening and must be able to understand, and willing to comply with the requirements of the protocol.

### Exclusion criteria

Patients were excluded if they had received treatment with any GnRH analogues within 6 months prior to screening, treatment with Danazol, Gestrinone or Cyproterone acetate within the 6 months prior to screening or anticipated requirement during the study, treatment with cyclical progestogens or combined oral contraceptives within one full menstrual cycle prior to screening, or anticipated requirement for these treatments during the study period and finally treatment with any other medication for CCPP (other than simple analgesics) within three months prior to screening.

Patients were also excluded if they had continuous or acyclic pelvic pain, metabolic bone disease, unexplained vaginal bleeding or any other medical condition that in the opinion of the investigator would impact upon the safety or efficacy of the study treatment or any study assessments.

The presence of an abnormal full blood count (FBC), liver or renal functions at screening or within the 6 months prior to screening or having a Bone mineral density (BMD) age adjusted T-Score of − 2 or below at the screening visit were also indications for exclusion from the study. Women were also excluded if they were receiving concomitant treatment with Coumarin or Indanedione derivatives or had a known contraindication, allergy or hypersensitivity to any of the test compounds or materials (including both Triptorelin® SR and Tibolone).

Finally, patients were excluded if they were pregnant or lactating, planning a pregnancy within 31 months of screening, unwilling to use adequate barrier contraception for the duration of the study, had received any investigational drug therapy within 30 days prior to the study, were scheduled to receive such a drug during the study period or had previously entered this study. Patients of child-bearing potential (i.e. who are not surgically sterile) must have a negative urine pregnancy test at the baseline visit.

Patients who fulfilled the inclusion/exclusion criteria and agreed to participate in the study attended for a screening visit at which the patient’s medical history was reviewed, vital signs were documented and blood samples were collected for routine haematology and biochemistry analysis. Any previous or concomitant medications were recorded. The patient was given a diary and instructed on its completion. Bone density was determined using a dual-energy x-ray absorptiometry (DEXA scan) conducted either at the screening visit or in the interval between the screening and baseline visits. Within two months of the screening visit, patients who fulfilled the inclusion/exclusion criteria returned to the clinic for the baseline visit. At this visit a physical check and urine pregnancy test were performed, vital signs were measured, pain and Health Related Quality of Life (HR-QoL) questionnaires were completed. Patients who were considered eligible for the trial received an injection of Triptorelin® SR 11.25 mg in the late luteal phase of the menstrual cycle, and were given enough Tibolone® 2.5 mg tablets to cover the time period until the following study visit. The diary given at the screening visit was collected and reviewed and patients were given a new diary card.

Patients returned for repeat Triptorelin injections every three months until month 21 at which point the last Triptorelin injection was administered. At these visits, patients were also dispensed additional amounts of Tibolone. Patients were required to return their used Tibolone packaging and any remaining pills at every study visit. These were checked to validate compliance. At each injection visit, the completed patient diary was collected and a further patient diary dispensed.

Follow-up assessments were conducted at 6, 12, 18 and 24 months following the baseline visit. An end of study follow-up evaluation was conducted six months after the end of the 24-month treatment period of the study (at month 30). At every follow-up visit HR-QoL questionnaires were given. Vital signs, bone density assessment DEXA and haematology and biochemistry analysis were repeated at months 12, 24 and 30. The physical examination was repeated at months 24 and 30. The reasons and numbers of drop outs over the course of the study is seen in Table 1. A Gant chart of procedure performed at each visit is seen in Table 2.

**Table 1:** Number and reason for drop outs at each visit

Clinical visit (n)	Drop out (n)
Screening (*n* = 31)	Failed screening (*n* = 2) Lost to follow up (*n* = 1) Withdrawal of consent (*n* = 1)
Baseline (*n* = 27)	No longer eligible (n = 1) Withdrawal of consent (*n* = 1)
3 months (*n* = 25)	Intolerable menopausal symptoms (*n* = 1) No improvement in symptoms (*n* = 2)
6 months (*n* = 22)	No improvement in symptoms (n = 1)
9 months (*n* = 21)	Wishing to conceive (*n* = 1) No improvement of symptoms (*n* = 1)
12 months (*n* = 19)	Wishing to conceive (n = 1) No improvement of symptoms (n = 1)
15 months (*n* = 17)	Intolerable menopausal symptoms (n = 2)
18 months (*n* = 15)	Intolerable menopausal symptoms (n = 1) Lost to follow up (n = 1)
24 months (*n* = 13)	No further drop outs

**Table 2:** Summary of procedures performed during the study visits. The study divided into three phases; screening, treatment and post-screening phase which distributed into 11 visits where each visit had certain evaluation measurements

	Screening phase	Treatment phase Triptorelin SR 11.25 mg injected 3-monthly starting at baseline. Tibolone 2.5 mg tablet taken daily from baseline.									Follow-up phase
	Visit 1 Screen	Visit 2^2^	Visit 3	Visit 4	Visit 5	Visit 6	Visit 7	Visit 8	Visit 9	Visit 10	Visit 11
Written informed consent	X										
Medical history	X										
History of CCPP	X										
Physical examination		X								X	X
Vital signs ^5^	X	X				X				X	X
Urine pregnancy test		X									
Review of Inclusion / Exclusion Criteria	X	X									
Enrolment in the study		X									
Bone density (DEXA)	X					X				X	X
CPG questionnaire		X		X		X		X		X	X
EHP-30 questionnaire		X		X		X		X		X	X
Blood samples for haematology and biochemistry	X					X				X	X
Triptorelin injection		X	X	X	X	X	X	X	X		
Dispense Tibolone		X	X	X	X	X	X	X	X		
Tibolone compliance checked			X	X	X	X	X	X	X	X	
Dispense patient diary	X	X	X	X	X	X	X	X	X	X	
Collect patient diary		X	X	X	X	X	X	X	X	X	X
Prior and concomitant medications	X	X	X	X	X	X	X	X	X	X	X
Adverse events		X	X	X	X	X	X	X	X	X	X
End of study details											X

1 Screening was conducted up to 2-months prior to the baseline visit. Screening and baseline visits were combined if the results of the DEXA scan were available for review prior to enrolment.

2 The baseline visit took place in the late luteal phase of the patient’s menstrual cycle.

### Outcomes measures

1. **Safety Outcomes:**

- Significant change in any of the parameters of the full blood count, liver and renal function tests over the course of the study.
- Change in the bone mineral density (BMD) as assessed by Dual Energy X-ray absorptiometry for both the lumbar spine (L1-L5) and proximal femur. For consistency, the patient’s scan was performed on the same machine for the duration of the study. Osteopaenia was defined a decrease in the T score by 1–2.5 SD while osteoporosis was defined a decrease in the T score by 2.5 SD or more.
- Occurrence of adverse events (AEs): All adverse events occurring over the course of the study were recorded. An adverse event was defined as any unfavourable medical episode occurring during the course of the study which may or may not be related to the study medications. Adverse events were classified as related or unrelated to the treatment medications. In addition, they were classified according to severity into:
  - Mild: symptoms do not alter patient’s healthy functioning.
  - Moderate: symptoms provide some degree of impairment to function, but are not hazardous.
  - Severe: symptoms are hazardous to well-being, serious impairment of function or incapacitation.
- Serious Adverse Events (SAEs): A serious adverse event was defined as any event which results in death or life-threatening events, or which results in patient hospitalisation or prolongation of existing hospitalisation. Any SAEs were reported immediately (within 24 h), independent of the circumstances or suspected cause, to the research nurse or the Principle Investigator.

1. **Efficacy Evaluations**

Two quality of life questionnaires were used to examine the efficacy of treatment and effect on Quality of life (QoL), the Chronic Pain Grade questionnaire and the Endometriosis Health Profile 30.

- Chronic pain grade (CPG)

This was developed in 1992 [21] and designed to assess the severity of chronic pain problems. The CPG is based on measures of pain intensity and pain-related disability and has been extensively validated [21, 22]. The CPG consists of seven items providing a score which enables chronic pain to be classified into one of four hierarchical categories based on pain intensity and disability:

• Grade I – low disability, low pain intensity.

• Grade II – low disability, high pain intensity.

• Grade III – high disability, moderately limiting.

• Grade IV – high disability, severely limiting.

In addition to the categorical grading scheme above, the CPG contains numerical self-rating scales for characteristic pain intensity and disability scores. At each assessment time point, patients were asked to consider historical pain over the past six months

- The Endometriosis Health Profile-30 (EHP-30):

The Endometriosis Health Profile-30 consists of a core questionnaire containing 30 items and five scales: pain, control and powerlessness, emotional well-being, social support, and self-image. There are also six additional modular scales: work, intercourse, relationship with children, the medical profession, treatment, and infertility. EHP-30 scale scores are standardised on a range of 0 to 100. The mean score per category and the global score were calculated. A lower score indicates better health status. The EHP-30 has been shown to be responsive to change in patients with endometriosis and is more sensitive to change than the generic questionnaire 36-item short form health survey (SF-36) [23].

The primary efficacy criterion was the change in Chronic Pain Intensity Score (CPI) at Month 12 in comparison with the Baseline visit while the secondary endpoints were changes in the individual assessment questionnaires at each visit compared to the baseline.

#### Sample size determination

Assuming that Chronic Pain Intensity Score (CPI) at Month 24 is the primary endpoint, in a previous 18-month study with Zoladex, a 5.6 decrease at month 18 from baseline was observed (SD = 2.08). Aiming to demonstrate 2 points decrease and assuming conservatively that the corresponding SD = 3, based on a two sided 5% alpha level and a power of 90%, 26 patients would need to be included (based on a paired t-test). Allowing for a dropout rate of 35%, a total of 40 patients were aimed to be included in the study.

### Statistical analysis

Anonymised data were analysed using the Statistical Package for Social Sciences (SPSS, version 22; Chicago, IL). Demographic data were expressed as mean (SD) or median (range) as appropriate. Within group analysis was performed using Wilcoxon signed-rank tests. A Bonferroni correction was used as appropriate. Chi-square analyses and Fisher’s exact tests were used to study categorical variables. *P* values of < 0.05 were considered significant.

## Results

### Study population

Between December 2008 and May 2016, 31 patients with CCPP were enrolled out of 193 approached for the study. Thirty-one patients were consented and of these 27 participants commenced the study. The mean (± SD) age was 33.35 (± 7.3) years and the mean (± SD) BMI was 27.42 (± 5.9) kg/m^2^. Seventy four percent of the study population had a diagnosis of endometriosis. Predominant presenting symptoms of the study population are seen in Table 3.

**Table 3:** Clinical characteristics of the study population

Predominant symptom	Percentage
Severe dysmenorrhea	85.2%
Moderate dysmenorrhea	14.8%
Severe dyspareunia	18.5%
Moderate dyspareunia	29.6%
Mild dyspareunia	3.7%

## Safety outcomes

### Haematological profile

All values for haematological parameters can been seen in Table 4. Results show comparison for values between baseline and 12 months, baseline and 24 months, 12 months and 24 months and between 24 and 30 months. Wilcoxon ranked test was used with Bonferroni correction (*p* <  0.0125).

**Table 4:** Comparison of haematological parameters at different points in the study. Values are expressed as median (range)

	Baseline	p*	12 months	p**	24 months	p***	30 months	p****
HCT	0.39(0.33–0.47)	0.008	0.42(0.36–0.47)	0.74	0.42(0.38–0.46)	*p* < 0.001	0.41 (0.37–0.46)	0.16
RBCs	4.48 (3.66–5.13)	0.008	4.67(3.66–5.13)	0.42	4.77(4.20–5.62)	P < 0.001	4.6 (4.14–5.23)	0.07
Neutrophils	4.8 (2.10–9.50)	0.042	4.1(1.40–6.86)	0.16	3.5(0.31–6.12)	*p* = 0.007	4.31(3–6.56)	0.18
Monocytes	0.54 (0.20–1.10)	0.009	0.45(0.24–0.70)	0.18	0.46(0.30–0.60)	*p* = 0.34	0.53 (0.30–0.91)	0.13

p* baseline vs 12 months.

p** 12 months vs 24 months.

p*** baseline vs 24 months.

p**** 24 months vs 30 months.

### Liver function tests

Compared to baseline, results at 24 months showed no significant changes in the mean (± SD) ALT {24 U/L (± 7.53) vs 28 U/L (± 21.84)} *p* = 0.05 or AST {22.77 U/L (± 4.75) vs 21 U/L (± 7.35) *p* = 0.33}. There was a significant transient increase in the ALT concentration at 12 months however the concentration still remained within the normal range {26.15 U/L (7.53), Bonferroni adjusted *p* <  0.0125}.

### Renal function tests

Compared to baseline, results at 24 months showed no significant changes in the mean (± SD) concentration of urea {4.73 mmol/L (± 0.88) vs 4.18 mmol/L (± 1.07) *p* = 0.67} or creatinine {68.31 mmol/L (± 12.9) vs 64.15 mmol/L (± .82) *p* = 0.23). There was also no significant change at any other point in the study.

### Bone mineral density

#### Lumbar spine

Although there was no evidence for a significant change in the median (range) T score for the lumber spine over the course of the study {baseline 0.1 (− 1.7–2.1), 12 months − 0.5 (0.0–1.0) and 24 months − 0.3 (0.0–1.0, (*p* = 0.9}, there was a significant increase in the prevalence of osteopenia by 12 months (Chi square = 19.71, *p* < .001).

Six participants started the study with evidence of osteopaenia. Data was available from 5 of these six participants at 12 months, 3 participants at 24 months and 2 participants at 30 months. Data from the two osteopaenic participants who completed the study showed that one remained osteopaenic and the other had developed osteoporosis by the 30-month visit. For other participants who started the study with normal BMD. One developed osteopaenia by 12 months and remained osteopaenic by 30 months. One developed osteopaenia by 24 months and remained osteopaenic by 30 months one more participant developed osteopaenia by 30 months.

#### Femoral region

There was no evidence for a significant change in the median (range) T score for the proximal femur over the course of the study {baseline 0.45 (− 1.4–2.4), 12 months 0.3 (0.0–1.0) and 24 months 0.4 (0.0–1.0), *p* = 0.9}. Four participants started the study with evidence of osteopaenia (2 of these participants also had osteopaenia of the lumbar spine). Only one of these participants completed the 30 month follow up and continued to be osteopaenic. The two other participants reached the 12 month follow up, where one was osteopaenic (the same participant had osteopaenia of the lumbar spine and developed osteoporosis of the lumbar spine by the completion of the study). The remaining participant had normal BMD. None of the participants who started the study with normal femoral BMD developed osteopaenia or osteoporosis.

Adverse events: Study specific adverse effects are listed in Table 5. There were no reported serious events related to the study medications.

**Table 5:** Adverse events that could be related to the treatment

Adverse event	Number of episodes	Categories of the adverse events	Number of patients affected
Vaginal bleeding	23	Reproduction	13
Depression	2	Psychiatric disorders	2
Loss of libido	2	Psychiatric disorders	2
Mood swings	1	Psychiatric disorders	1
Hot flush	10	Vascular	9
Night sweats	2	Skin disorders	2
Pain at injection site	2	General Disorders	1

### Efficacy assessments

#### Health-related quality of life (EHP-30 questionnaire)

##### Core questionnaire

- Pain domain: There was a significant improvement in the median (range) of the pain score at the 24 months follow up compared to baseline {7.9 (0–61.4) and 56.8 (.0–95.5)} respectively (Z = 3.301, *p* <  0.001). This was followed by a significant deterioration after discontinuation of treatment at the 30-month visit {40.9 (0–75)} (Z = − 2.51, *p* = 0.009).
- Powerlessness domain: There was a significant improvement in the median (range) score at 24 months compared to baseline {14.8 (0–78.5) and 75 (0–100) respectively} (Z = − 3.16, *p* <  0.001). This again significantly deteriorated after discontinuation of treatment at the 30-month follow up {54.2 (0–91.7)} (Z = − 2.45, *p* = 0.006).
- Emotional well-being domain. There was a significant improvement in the median (range) score by 24 months when compared to baseline {8.3 (0–50) and 54.2 (0–100) respectively} (Z = 3.301, *p* <  0.001). This again significantly deteriorated after discontinuation of treatment at the 30-month follow up {45.8 (0–70.8)} (Z = − 2.51, *p* = 0.009)
- Social support and self-image domains: There was a significant improvement by 24 months when compared to baseline {(6.2 (0–75) and 50 (0–93.6)} respectively (Z = − 2.79, *p* = 0.003). There was no evidence for a significant change in the self-image domain at 24 months compared with baseline {(37.5 (0–100) and 58.33 (0–100) respectively} (Z = − 1.92, *p* = 0.054). There was no significant change in either of these domains after discontinuation of treatment by the 30-month follow up.

##### Modular questionnaire

- Work domain: There was a significant improvement in mean (median) score by 24 months compared to baseline {0 (0–60) and 45 (0–85)} respectively (Z = − 2.54, *p* = 0.008). There was a significant deterioration after discontinuation of treatment at the 30-month follow up {40 (0–90)} (Z = − 2.21, *p* = 0.03).
- Other domains: There was no evidence for a significant change in the “feeling of frustration with the medical profession”, “quality of sexual intercourse” “relationship with children” and “infertility” domains by the end of the study

Changes in EPH-30 domain scores are seen in Table 6.

**Table 6:** Comparison of baseline EPH-30 domain scores at other points of follow up (baseline versus 6, 12 and 18 month visits). Values are expressed as median (range) with respective *p* values

	Baseline	6 m	p	12 m	p	18 m	p
Pain domain	56.8 (0–95.5)	19.3(0–90.9)	< 0.001	11.4 (0–72.7)	< 0.001	9.08(0–56.8)	< 0.001
Control and powerlessness	75 (0–100)	16.7(0–100)	< 0.001	16.7(0–100)	< 0.001	14.3(0–79.2)	< 0.001
Emotional well-being	54.2 (0–100)	18.7(0–95.8)	0.01	25.4(0–75)	< 0.001	20.8 (0–68.3)	< 0.001
Social support	50 (0–93.6)	15.6 (0–39.5)	0.03	12.5(0–93.7)	< 0.001	12.5(0–100)	0.006
Self-image	58.3 (0–100)	22.5(0–100)	0.004	33.3(0–100)	< 0.001	33.3(0–91.7)	0.001
Work domain	45 (0–85)	12.5(0–80)	0.91	0 (0–80)	0.004	0 (0–70)	0.001

#### Chronic pain grade questionnaire

Over the course of the study and compared to baseline, there was a significant improvement in the pain intensity, disability and pain grade scores (Table 7).

**Table 7:** Comparison of baseline CPG domain scores versus 6, 12 and 18 month visits. Values are expressed as median (range) with respective *p* values

	Baseline	6 m	p	12 m	p	18 m	p	24 m	p
Pain intensity	66.6 (30–93)	39.9 (0–83.3)	0.001	30 (0–76.7)	< 0.001	30 (0–73.3)	p < 0.001	13.33 (0–36.3)	< 0.001
Disability	60 (16–113)	16.7 (0–93.3)	0.002	0 (0–66.7)	< 0.001	0 (0–63.3)	*p* < 0.001	0 (0–63)	0.001
Pain grade	3 (1–4)	1 (0–4)	0.004	0 (0–4)	0.001	0 (0–4)	p < 0.001	0 (0–4).	0.002

At 30 months and after discontinuation of treatment there was a significant deterioration in the median (range) pain intensity and disability scores (compared to 24 months) {56.66 (6–80), *p* = 0.001 and 30 (0–66.7), p = 0.001} respectively.

## Discussion

The licenced use of GnRHa for endometriosis is limited to six months in the UK, although in many cases they are needed for longer durations, particularly when surgical intervention is deemed non-feasible. The result of these restrictions is that for those women who gain benefit from the agonist, treatment must be withdrawn almost as soon as it has become effective. This is a cause of frustration and for both patients and clinicians. New data on long term safety, particularly with respect to impact on bone mineral density, are therefore needed.

Although many studies have examined the efficacy of GnRHa for CCPP and endometriosis over periods of up to six months [24], 12 months [18] and 18 months [25], to the best of our knowledge this is the first study to provide data regarding both safety and efficacy of GnRHa for a long-term 24-month period in women with CCPP.

The study design with longitudinal six monthly follow-ups allowed for the gathering of detailed information regarding changes that occur beyond the usual licensed use, the temporal relationship of these changes as well as an assessment of overall compliance with long term therapy. The study also provides information regarding quality of life and safety parameters up to 6 months after completing a 24-month therapeutic course.

The first result of this study was that there was a significant improvement in pain related QoL parameters that continued throughout the duration of the study but significantly deteriorated after cessation of the medication. The effects of CCPP and endometriosis on QoL have been reported in many previous studies [26–30].

We used the well-validated quality of life instrument EPH-30 to assess the benefits of long term GnRHa on patient symptoms. We found that GnRHa plus addback treatment led to a significant improvement in quality of life and in pain scores by six months of therapy. However, other modules, including those assessing relationship with children, sexual intercourse and infertility did not show a significant change. This may be due to the fact that not all patients had children, were sexually active or were trying to conceive. Patients wishing to conceive were excluded from the study as GnRHa inhibit ovulation rendering the patient temporarily infertile.

Other modules, such as the patients’ feelings of frustration about the treatment and about the medical profession, did not show significant improvement. This could be due to the chronic nature of the condition which may have influenced the patients’ feelings regarding possible long-term cure and creating an element of pessimism regarding full cure [31]. Physicians treating patients with CCPP should be aware of the psychological dimensions that often form a large part of the patient’s problems, and consider referral for counselling regarding long-term prognosis and management, particularly after discontinuation of GnRHa treatment.

Regarding the safety of administration of GnRH analogues and add back therapy beyond the licensed 6-month period, we found no evidence for a significant adverse effect on haematological parameters, liver or renal functions associated with 24 months of treatment. The only significant change was an increase in RBC and haematocrit which is probably as a result of the temporary state of pseudo-menopause leading to the cessation of menstruation. Cessation of menses may also have contributed to the improvement in the quality of life parameters seen in this study, since many patients initially presented with dysmenorrhoea so it would be expected that the cessation of menstruation would have a positive effect.

Although ALT concentrations did show a significant change after 12 months of the study, this change seemed to be temporary as it resolved by the 18 month follow up. Furthermore, despite this change, ALT concentrations always remained within the normal range and there were no observed changes in other liver enzymes during the study. Again, this transient change in ALT may be due to the pseudo-menopausal state, as similar changes in ALT concertation have previously been reported in menopausal females [32].

The principal concern when using long-term GnRHa relates to loss of BMD [18, 25]. This study suggested a risk of deterioration in BMD with prolonged use of GnRH despite the use of Tibolone add back therapy. There are several important points to note in this regard. Firstly, a significant number of patients started the study with already compromised BMD (i.e. osteopaenia) and it is one of these patients who developed osteoporosis by the end of the study. For those who developed osteopenia for the first time during the study (14%), it is important to note that the osteopaenia persisted at the 30 month follow up (i.e. 6 months after discontinuation of the GnRH therapy). Furthermore, since some of these patients did not complete the study or were lost to follow up, it is unclear whether any of them had improved, stabilised or deteriorated BMD at the 30 months follow up. It is of course expected that any loss of bone mineral density was mitigated by the use of tibolone. Without a control arm where tibolone was not prescribed, it would be impossible to quantify such a benefit. Such a study design however would be ethically questionable.

Finally, the risk of developing osteoporosis with prolonged GnRHa therapy is often less than the significant additional surgical risk associated with operating on such patients. In many cases surgically removing the ovaries (with or without hysterectomy), when appropriate, would subject the patient to similar risks regarding BMD even if HRT were used. It is therefore important to weigh the risks of both approaches. This study therefore emphasises the importance of regular follow up of BMD in patients on prolonged GnRH therapy. Furthermore, a baseline scan performed prior to starting treatment may screen for patients who already have compromised BMD and therefore are at higher risk and require closer follow up. It would also be of interest to examine the use of higher dose oestrogen add back regimens in future studies, since this approach may offer better protection against loss of BMD without compromising the efficacy of the GnRHa therapy on pain. It would also be interesting to examine the use of different add-back therapy drug delivery systems such as the use of oestrogen patches which are known to be associated with increased oestrogen plasma concentration due to the avoidance of the hepatic first-pass effect [33].

No Serious Adverse Events occurred during the study. Some Adverse Events did occur, which may have affected compliance and contributed to the dropout rate. Nevertheless, these AEs occurred in only few patients and for only a few episodes. It is difficult to ascertain whether commonly reported Adverse Events such as headaches were the result of the medication or due to some other non-related condition [34]. It has been reported that the mere presence of endometriosis may be associated with such symptoms as result of hormonal fluctuations [35–37].

The current study is not without limitations. Although we aimed to recruit a larger number of women, this proved to be logistically difficult. Despite the large number who were screened (193 women), only 31 provided consent to participated and of these 27 started the study. The study also had a significant drop out rate. These issues however were expected due to the very nature of the condition, the length of the study and the demographics of the study population. For example, it is expected that there would be fluctuations in severity of CCPP which may affect compliance. Furthermore, in such a relatively young group priorities may shift. For example, a desire for fertility or a change in work circumstances requiring a move to another location as well as the psychological effects of long term pelvic pain and the need of long term therapy may affect patients’ motivation and compliance with treatment. Nevertheless, we believe that our results are a benefit to clinicians who wish to use this approach since it informs them that (a) the medications will not work in 100% of women who start treatment and (b) frequent follow up and reinforcement is necessary to maintain good compliance with treatment. Involvement of the multidisciplinary team with input from nurse practitioners and psychologists is helpful here.

Secondly, additional confounding factors influencing the rate of bone metabolism such as lifestyle (exercise, alcohol, smoking and caffeine consumption) and genetic factors were not analysed in this study. Furthermore, in future studies when analysing changes in the BMD over time, values should be adjusted for advancement of bone age over the course of the study (30 months = 2.5 years) to account for the excepted 1% annual loss in BMD [38].

Self-reporting questionnaires were used in this study and although these were well validated, self-reporting has disadvantages due to the influence of personal attitudes and beliefs particularly when reporting on domains such as sexual behaviour. Finally, further studies are now needed to explore safety and efficacy beyond 24 months of therapy in order to provide women with an even longer-term solution which may truly influence their attitude.

In conclusion, the use of long- term Triptorelin plus Tibolone as add-back therapy in pre-menopausal women suffering from CCPP and endometriosis does not appear to be associated with significant serious adverse events apart from the possibility of deterioration in the BMD that needs to be monitored. This mode of therapy appears to be highly effective in pain relief and in improving quality of life over a 24-month period. Further prospective studies are needed to assess other means of protecting BMD during long-term GnRHa treatment and to use metabolic parameters such as serum bone alkaline phosphatase (sBAP), serum N-terminal telopeptide of type I collagen (NTX), in addition to the DEXA scan to determine the optimum treatment period for individual patients. We hope that the findings of this study will help change the existing licencing restrictions on the duration of GnRHa therapy and help shape National and International guidelines regarding the treatment of this group of women who often have limited alternate options.

## Data Availability

Data available on request from the author but subject to permission from the sponsor “Sheffield Teaching Hospitals.

## References

[1] Latthe P, Latthe M, Say L, Gulmezoglu M, Khan KS (2006). WHO systematic review of prevalence of chronic pelvic pain: a neglected reproductive health morbidity. BMC Public Health.

[2] Brawn J, Morotti M, Zondervan KT, Becker CM, Vincent K (2014). Central changes associated with chronic pelvic pain and endometriosis. Hum Reprod Update.

[3] Triolo O, Lagana AS, Sturlese E (2013). Chronic pelvic pain in endometriosis: an overview. J Clin Med Res.

[4] Giamberardino MA, Tana C, Costantini R (2014). Pain thresholds in women with chronic pelvic pain. Curr Opin Obstet Gynecol.

[5] Morotti M, Vincent K, Brawn J, Zondervan KT, Becker CM (2014). Peripheral changes in endometriosis-associated pain. Hum Reprod Update.

[6] Hoffman D (2015). Central and peripheral pain generators in women with chronic pelvic pain: patient centered assessment and treatment. Curr Rheumatol Rev.

[7] Stratton P, Khachikyan I, Sinaii N, Ortiz R, Shah J (2015). Association of chronic pelvic pain and endometriosis with signs of sensitization and myofascial pain. Obstet Gynecol.

[8] Moore JS, Gibson PR, Perry RE, Burgell RE (2017). Endometriosis in patients with irritable bowel syndrome: specific symptomatic and demographic profile, and response to the low FODMAP diet. Aust N Z J Obstet Gynaecol.

[9] Doggweiler R, Whitmore KE, Meijlink JM, Drake MJ, Frawley H, Nordling J, Hanno P, Fraser MO, Homma Y, Garrido G (2017). A standard for terminology in chronic pelvic pain syndromes: a report from the chronic pelvic pain working group of the international continence society. Neurourol Urodyn.

[10] Saridogan E (2017). Adolescent endometriosis. Eur J Obstet Gynecol Reprod Biol.

[11] Won HR, Abbott J (2010). Optimal management of chronic cyclical pelvic pain: an evidence-based and pragmatic approach. Int J Women's Health.

[12] Munoz-Hernando L, Munoz-Gonzalez JL, Marqueta-Marques L, Alvarez-Conejo C, Tejerizo-Garcia A, Lopez-Gonzalez G, Villegas-Munoz E, Martin-Jimenez A, Jimenez-Lopez JS (2015). Endometriosis: alternative methods of medical treatment. Int J Women's Health.

[13] Millar RP, Newton CL (2013). Current and future applications of GnRH, kisspeptin and neurokinin B analogues. Nat Rev Endocrinol.

[14] Ubuka T, Son YL, Tsutsui K (2016). Molecular, cellular, morphological, physiological and behavioral aspects of gonadotropin-inhibitory hormone. Gen Comp Endocrinol.

[15] https://bnf.nice.org.uk/drug/triptorelin.html - indicationsAndDoses.

[16] Kim NY, Ryoo U, Lee DY, Kim MJ, Yoon BK, Choi D (2011). The efficacy and tolerability of short-term low-dose estrogen-only add-back therapy during post-operative GnRH agonist treatment for endometriosis. Eur J Obstet Gynecol Reprod Biol.

[17] Zito G, Luppi S, Giolo E, Martinelli M, Venturin I, Di Lorenzo G, Ricci G (2014). Medical treatments for endometriosis-associated pelvic pain. Biomed Res Int.

[18] DiVasta AD, Feldman HA, Sadler Gallagher J, Stokes NA, Laufer MR, Hornstein MD, Gordon CM (2015). Hormonal add-Back therapy for females treated with gonadotropin-releasing hormone agonist for endometriosis: a randomized controlled trial. Obstet Gynecol.

[19] Zheng Q, Mao H, Xu Y, Zhao J, Wei X, Liu P (2016). Can postoperative GnRH agonist treatment prevent endometriosis recurrence? A meta-analysis. Arch Gynecol Obstet.

[20] Pinto-Almazan R, Segura-Uribe JJ, Farfan-Garcia ED, Guerra-Araiza C (2017). Effects of Tibolone on the central nervous system: clinical and experimental approaches. Biomed Res Int.

[21] Von Korff M, Ormel J, Keefe FJ, Dworkin SF (1992). Grading the severity of chronic pain. Pain.

[22] Smith BH, Penny KI, Purves AM, Munro C, Wilson B, Grimshaw J, Chambers WA, Smith WC (1997). The chronic pain grade questionnaire: validation and reliability in postal research. Pain.

[23] Jones G, Kennedy S, Barnard A, Wong J, Jenkinson C (2001). Development of an endometriosis quality-of-life instrument: the endometriosis health Profile-30. Obstet Gynecol.

[24] Agarwal SK, Daniels A, Drosman SR, Udoff L, Foster WG, Pike MC, Spicer DV, Daniels JR (2015). Treatment of endometriosis with the GnRHa Deslorelin and add-Back estradiol and supplementary testosterone. Biomed Res Int.

[25] Al-Azemi M, Jones G, Sirkeci F, Walters S, Houdmont M, Ledger W (2009). Immediate and delayed add-back hormonal replacement therapy during ultra long GnRH agonist treatment of chronic cyclical pelvic pain. BJOG.

[26] Nnoaham KE, Hummelshoj L, Webster P, d'Hooghe T, de Cicco NF, de Cicco NC, Jenkinson C, Kennedy SH, Zondervan KT (2011). World endometriosis Research Foundation global study of Women's health c: impact of endometriosis on quality of life and work productivity: a multicenter study across ten countrie**s**. Fertil Steril.

[27] Souza CA, Oliveira LM, Scheffel C, Genro VK, Rosa V, Chaves MF, Cunha Filho JS (2011). Quality of life associated to chronic pelvic pain is independent of endometriosis diagnosis--a cross-sectional survey. Health Qual Life Outcomes.

[28] Tripoli TM, Sato H, Sartori MG, de Araujo FF, Girao MJ, Schor E (2011). Evaluation of quality of life and sexual satisfaction in women suffering from chronic pelvic pain with or without endometriosis. J Sex Med.

[29] Jia SZ, Leng JH, Shi JH, Sun PR, Lang JH (2012). Health-related quality of life in women with endometriosis: a systematic review. J Ovarian Res.

[30] Kiykac Altinbas S, Bayoglu Tekin Y, Dilbaz B, Dilbaz S (2015). Evaluation of quality of life in fertile Turkish women with severe endometriosis. J Obstet Gynaecol.

[31] Cox H, Henderson L, Andersen N, Cagliarini G, Ski C (2003). Focus group study of endometriosis: struggle, loss and the medical merry-go-round. Int J Nurs Pract.

[32] Matsui S, Yasui T, Kasai K, Keyama K, Kato T, Uemura H, Kuwahara A, Matsuzaki T, Irahara M (2016). Changes of liver enzymes and triglyceride during the menopausal transition in Japanese women. J Obstet Gynaecol.

[33] Ramachandran C, Fleisher D (2000). Transdermal delivery of drugs for the treatment of bone diseases. Adv Drug Deliv Rev.

[34] Jensen R, Stovner LJ (2008). Epidemiology and comorbidity of headache. Lancet Neurol.

[35] Ferrero S, Pretta S, Bertoldi S, Anserini P, Remorgida V, Del Sette M, Gandolfo C, Ragni N (2004). Increased frequency of migraine among women with endometriosis. Hum Reprod.

[36] Karp BI, Sinaii N, Nieman LK, Silberstein SD, Stratton P (2011). Migraine in women with chronic pelvic pain with and without endometriosis. Fertil Steril.

[37] MacGregor EA (2009). Migraine headache in perimenopausal and menopausal women. Curr Pain Headache Rep.

[38] Nguyen TV, Sambrook PN, Eisman JA (1998). Bone loss, physical activity, and weight change in elderly women: the Dubbo osteoporosis epidemiology study. J Bone Miner Res.

